# Divergence of mechanistic pathways mediating cardiovascular aging and developmental programming of cardiovascular disease

**DOI:** 10.1096/fj.201500057

**Published:** 2016-03-01

**Authors:** Beth J. Allison, Joepe J. Kaandorp, Andrew D. Kane, Emily J. Camm, Ciara Lusby, Christine M. Cross, Rhianon Nevin-Dolan, Avnesh S. Thakor, Jan B. Derks, Jane L. Tarry-Adkins, Susan E. Ozanne, Dino A. Giussani

**Affiliations:** * Department of Physiology Development and Neuroscience, University of Cambridge, Cambridge, United Kingdom;; † Perinatology, University Medical Center, Utrecht, The Netherlands; and; ‡ Metabolic Research Laboratories and Medical Reseach Council (MRC) Metabolic Diseases Unit, Wellcome Trust–MRC Institute of Metabolic Science, Addenbrookes Hospital, Cambridge, United Kingdom

**Keywords:** cell senescence, fetal hypoxia, oxidative stress, allopurinol, xanthine oxidase

## Abstract

Aging and developmental programming are both associated with oxidative stress and endothelial dysfunction, suggesting common mechanistic origins. However, their interrelationship has been little explored. In a rodent model of programmed cardiovascular dysfunction we determined endothelial function and vascular telomere length in young (4 mo) and aged (15 mo) adult offspring of normoxic or hypoxic pregnancy with or without maternal antioxidant treatment. We show loss of endothelial function [maximal arterial relaxation to acetylcholine (71 ± 3 *vs.* 55 ± 3%) and increased vascular short telomere abundance (4.2–1.3 kb) 43.0 ± 1.5 *vs.* 55.1 ± 3.8%) in aged *vs.* young offspring of normoxic pregnancy (*P* < 0.05). Hypoxic pregnancy in young offspring accelerated endothelial dysfunction (maximal arterial relaxation to acetylcholine: 42 ± 1%, *P* < 0.05) but this was dissociated from increased vascular short telomere length abundance. Maternal allopurinol rescued maximal arterial relaxation to acetylcholine in aged offspring of normoxic or hypoxic pregnancy but not in young offspring of hypoxic pregnancy. Aged offspring of hypoxic allopurinol pregnancy compared with aged offspring of untreated hypoxic pregnancy had lower levels of short telomeres (vascular short telomere length abundance 35.1 ± 2.5 *vs.* 48.2 ± 2.6%) and of plasma proinflammatory chemokine (24.6 ± 2.8 *vs.* 36.8 ± 5.5 pg/ml, *P* < 0.05). These data provide evidence for divergence of mechanistic pathways mediating cardiovascular aging and developmental programming of cardiovascular disease, and aging being decelerated by antioxidants even prior to birth.—Allison, B. J., Kaandorp, J. J., Kane, A. D., Camm, E. J., Lusby, C., Cross, C. M., Nevin-Dolan, R., Thakor, A. S., Derks, J. B., Tarry-Adkins, J. L., Ozanne, S. E., Giussani, D. A. Divergence of mechanistic pathways mediating cardiovascular aging and developmental programming of cardiovascular disease.

Cardiovascular disease is the leading cause of death today, imposing a staggering burden on nearly every country’s health and wealth (1, 2). Across the world, heart disease results in 1 in 3 deaths per year and the economic load amounts to over £30 billion annually in the United Kingdom and over $130 billion per year in the United States and Canada (3, 4). Consequently, there is great interest in identifying therapeutic targets against risk factors promoting cardiovascular disease. Such risk factors include established concepts, such as aging (5), and more recently accepted ideas, such as developmental programming. Overwhelming evidence derived from human epidemiology and experimental studies in animal models now shows that adverse conditions during pregnancy increases susceptibility to cardiovascular disease in later life (6–8).

Interestingly, cardiovascular degeneration associated with normal aging and developmental programming of cardiovascular disease share a number of common features. These include loss of endothelial-dependent vasodilation in peripheral resistance circulations (7, 9–13) and shortened telomere length (14, 15). This raises the possibility that cardiovascular degeneration associated with normal aging and developmental programming of cardiovascular disease may share common mechanistic pathways. Indeed, many studies have suggested that developmental programming of disease may be a form of accelerated aging (16–21).

One candidate mechanism involved in both cardiovascular aging and developmental programming of cardiovascular disease is oxidative stress. Harman’s free radical theory of aging (22), which stated that inappropriate accumulation of free radicals was linked with cumulative cell damage, has stood the test of time. Since then, numerous reports have linked aging with enhanced oxidative/inflammatory stress and/or impaired antioxidant defenses (23–25). Similarly, many studies have reported that maternal treatment with antioxidants in complicated pregnancy protects against programmed cardiometabolic dysfunction in later life (11, 16, 26–31).

Although aging and a suboptimal intrauterine environment are independent risk factors for cardiovascular dysfunction in later life, investigation of their interrelationship has been restricted to few studies (13, 32). Furthermore, whether maternal antioxidant therapy protects against cardiovascular disease in the young adult as well as the aged offspring of healthy or suboptimal pregnancy is not known. The most common feature of suboptimal pregnancy is chronic fetal hypoxia (7, 12, 33), and a powerful pro-oxidant mechanism stimulated by chronic hypoxia is activation of the xanthine oxidase pathway (34, 35). Therefore, in this study, we have used an established rodent model of programmed cardiovascular dysfunction to test the interrelated hypotheses: *1*) that developmental hypoxia leads to an earlier loss of endothelial function associated with accelerated vascular telomere shortening in young adult offspring, and *2*) that maternal treatment with the xanthine-oxidase inhibitor allopurinol protects against cardiovascular dysfunction in young and aged adult offspring of hypoxic pregnancy by maintaining vascular telomere length. The young adult offspring were investigated at 4 mo and the aged adult offspring at 15 mo.

## MATERIALS AND METHODS

Experiments were carried out under the United Kingdom Animals (Scientific Procedures) Act 1986 and approved by the University of Cambridge Animal Welfare and Ethics Committee. Wistar rat pregnancies were established as described (27, 29, 36). On d 6 of pregnancy (term is 21 d), rats were randomly divided into 4 groups (*n* = 20 per group): control or hypoxic pregnancy, with or without maternal treatment with allopurinol (30 mg/kg/d in jelly). This dose and route of treatment with allopurinol crosses the placenta and inhibits xanthine oxidase activity in the maternal, placental, and fetal tissues (37). Hypoxic pregnancy was started on d 6 of gestation as exposure prior to this point markedly enhances pregnancy loss (28). Pregnancies undergoing hypoxia were maintained at a constant inspired fraction of oxygen of 13% inside a polyvinylchloride isolator, which was fed compressed air and nitrogen from a nitrogen generator to the required inspirate mixture (27, 28, 36). The isolator could hold 9 rat cages at any one time and it contained a transfer box, which permitted cages to be exchanged for clean ones without losing the hypoxic environment. The environment within the hypoxic isolator was changed 12–20 times per hour; it was quiet and tranquil and similar to that provided by cages in which normoxic pregnancies were maintained. These were individually ventilated and both normoxic cages and the hypoxic isolator were housed in the same room with a controlled 12 h light:dark cycle (27, 28, 36). Litters were allowed to deliver spontaneously. Following determination of birth weight, litter size, and the sex of the pups (anogenital distance), litters were culled to 3 males and 3 females to standardize nutritional access and maternal care. To control for sex differences, only male offspring were studied. At 4 and 15 mo, following weighing, 1 male from each litter per outcome variable underwent euthanasia. Femoral arteries were isolated and mounted for *in vitro* wire myography (*n* = 8/group). Descending aortas were frozen for molecular analysis of telomere shortening (*n* = 8/group), and blood samples were taken (*n* = 6/group).

### *In vitro* wire myography

Second-order femoral arteries were mounted on a 4-chamber small-vessel wire myograph (Multi Wire Myograph System 610M; DMT, Aarhus, Denmark) (38). Relaxant responses to methacholine (10^−10^–10^−4^ mM) were determined after precontraction with phenylephrine (submaximal). Concentration–response curves were analyzed using an agonist–response best-fit line. The maximal relaxant response was expressed as percentage of the contraction induced by phenylephrine and the vascular sensitivity was expressed as pD_2_ (−logEC_50_) (38).

### Measurement of telomere length

The descending aorta was removed and snap frozen in liquid nitrogen. The entire tissue sample was powdered on dry ice, and then nonsheared high molecular weight DNA was isolated from the finely powdered aorta sample using the phenol/chloroform/isoamyl alcohol DNA extraction procedure as detailed elsewhere (39). DNA integrity and quantity were determined using agarose gel electrophoresis and spectrophotometrically (Nanodrop; Nanodrop Technologies, Wilmington, DE, USA). High molecular weight DNA (1.2 µg) was digested with *Hinf1* and *Rsa1* restriction enzymes for 2 h at 37°C from collected descending aortas. The restricted DNA samples were quenched with 5 × SDS loading buffer (Roche Diagnostics, Mannheim, Germany) and loaded onto agarose gels containing SYBR safe stain (Invitrogen, Paisley, United Kingdom). DNA was separated using pulse field gel electrophoresis, for 7.5 h at 6 V/cm at a switching time of 1–30 s. The gels were checked for nonspecific degradation of an undigested DNA control and complete digestion of the enzyme-restricted DNA and visualized under UV light (Gel Doc, Syngene, Cambridge, United Kingdom). The separated DNA fragments were transferred to nylon membrane (Roche Diagnostics) by Southern blott using a vacuum blotter (Biorad, Hemel Hempstead, United Kingdom) for 90 min. The transferred DNA was cross-linked onto the membrane using a UV cross-linker (Stratagene, La Jolla, CA, USA). Telemeric repeat length was determined using a commercial method of chemiluminescent detection; TeloTAGGG telomere length assays (Roche Diagnostics) according to manufacturer’s instructions. Molecular weight markers on each gel were a high range Pulsed Field Gel marker (New England Biolabs, Ipswich, MA, USA) and dioxygenin (low-range) molecular weight marker (Roche Diagnostics). Standard undigested and digested genomic samples of DNA from a 4-mo-old control animal were also included on each gel to verify digestion efficiency. Telomere signals were analyzed using Adobe Photoshop (Adobe Systems Inc., San Jose, CA, USA) and MacBas software (Fujifilm UK, Bedford, United Kingdom). Telomere length was determined as the percentage intensity (% telomere length) of the telomeric signal in four molecular size regions, as defined by molecular weight markers (40). Discrete grid squares were placed around the telomeric smear according to the following molecular weights: 145–48.5, 48.5–8.6, 8.6–4.2, and 4.2–1.3 kb. The percentage intensity in each molecular weight range was measured (% intensity = intensity of a defined region − background × 100/total lane intensity − background).

### Plasma analysis

To determine possible changes in circulating inflammatory markers, plasma concentration of the cytokines/chemokines IFN-γ, IL-5, C-reactive protein, and proinflammatory chemokine [keratinocyte chemoattractant/growth-regulated oncogene (KC/GRO), the rodent equivalent of human IL-8] (41) were measured using the Multiplex MAP Magnetic bead-based immunoassay kits (Millipore Corp., Billerica, MA, USA) at the Core Biochemical Assay Laboratory, Cambridge, United Kingdom.

### Data and statistical analyses

The experimental and statistical design was stringent to account for sex differences and within litter variation. Comparisons of variables derived from more than 1 offspring per litter, such as birth weight and birth characteristics were performed using mixed linear model analysis. Other comparisons were of outcome variables derived from only 1 male offspring per litter per experimental group. These comparisons were assessed using a 2-way ANOVA with the Tukey *post hoc* test. For all comparisons, significance was accepted when *P* < 0.05.

## RESULTS

### Effects of aging

**Figure 1.**
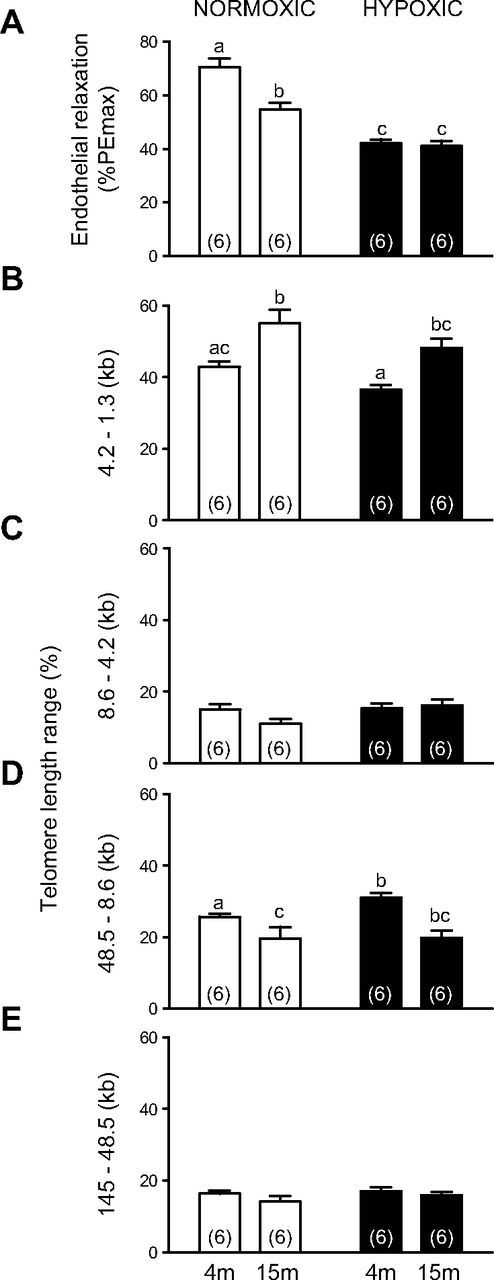
Effects of aging and developmental hypoxia on endothelial function and vascular telomere length. Values are means ± sem for the femoral artery maximal dilator response to methacholine (*A*, endothelial relaxation) expressed as a percentage of the phenylephrine-induced maximal constriction (%PEmax) and for the frequency (%) of aortic telomere length ranges (*B*, 4.2–1.3 kb; *C*, 8.6–4.2 kb; *D*, 48.5–8.6 kb; and *E*, 145–48.5 kb) in 4- and 15-mo-old offspring of normoxic (white bars) or of hypoxic (black bars) pregnancy. Numbers of animals for each group are in brackets. Bars with different letters are significantly (*P* < 0.05) different (2-way ANOVA and *post hoc* Tukey’s test).

Endothelial vasodilator function was investigated using *in vitro* wire myography of second-order femoral arterial segments. Vascular telomere shortening was investigated by determining aortic telomere length by Southern blot. Femoral arterial segments isolated from aged (15 mo) relative to young adult (4 mo) offspring of normoxic pregnancy had impaired relaxant responses to the acetylcholine analog methacholine following preconstriction with phenylephrine (**Fig. 1*A***). Vascular tissue isolated from aged relative to young adult offspring of normoxic pregnancy also showed an increased frequency of short telomeres (Fig. 1*B*).

### Effects of developmental hypoxia

Offspring of hypoxic relative to offspring of normoxic pregnancy showed impaired femoral endothelial relaxation already at 4 mo of young adult age (Fig. 1*A*). The vascular relaxant deficit in young adult offspring of hypoxic pregnancy at 4 mo was significantly greater than that measured in aged offspring of normoxic pregnancy at 15 mo, but was not exacerbated by aging (Fig. 1*A*). However, this accelerated loss of endothelial function in young adult offspring of hypoxic pregnancy was not associated with an earlier increase in the frequency of short telomeres (Fig. 1*B*) or an earlier decrease in the frequency of long telomeres (Fig. 1*C–E*) in vascular tissue relative to offspring of normoxic pregnancy.

### Effects of maternal treatment with allopurinol

**Figure 2.**
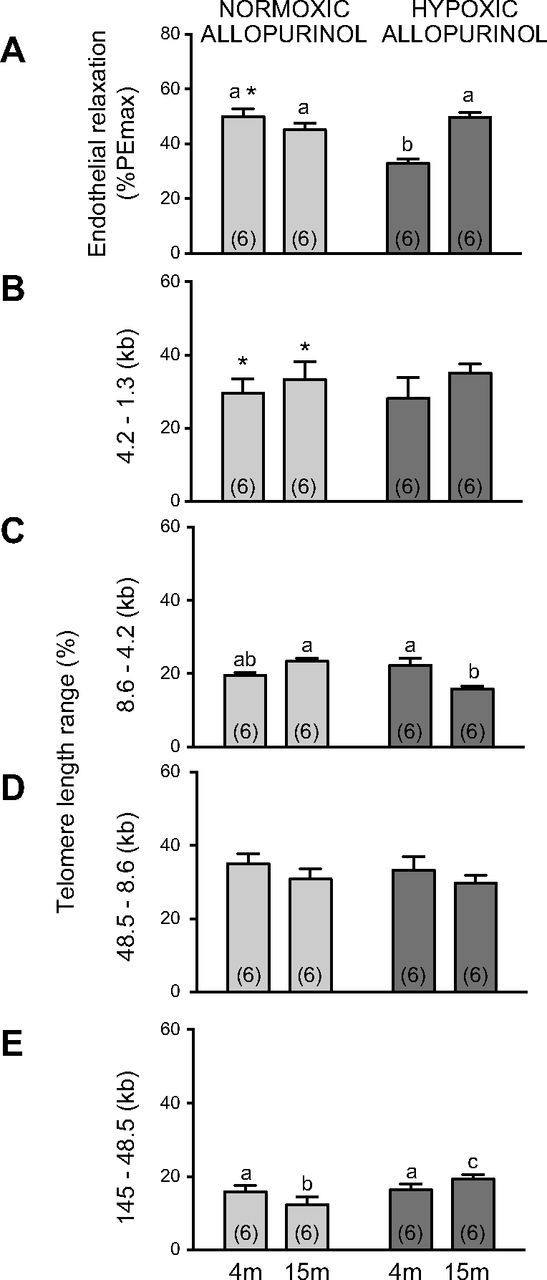
Effects of maternal treatment with allopurinol on endothelial function and vascular telomere length in young and aged offspring of normoxic or hypoxic pregnancy. Values are means ± sem for the femoral response to methacholine (*A*, endothelial relaxation) expressed as a percentage of the phenylephrine-induced maximal constriction (%PEmax) and for the frequency (%) of aortic telomere length ranges (*B*, 4.2–1.3 kb; *C*, 8.6–4.2 kb; *D*, 48.5–8.6 kb; and *E*, 145–48.5 kb) in 4- and 15-mo-old offspring of normoxic (stippled bars) or of hypoxic (gray bars) pregnancy following maternal treatment with allopurinol. Numbers of animals for each group are in brackets. Bars with different letters are significantly different (*P* < 0.05). *P < 0.05 offspring of treated *vs.* untreated pregnancy (2-way ANOVA and *post hoc* Tukey’s test).

When comparing treated *vs.* untreated pregnancy, maternal treatment with allopurinol significantly decreased the magnitude of endothelial relaxation in young adult offspring of normoxic pregnancy (Fig. 1*A* *vs*. **Fig. 2*A***; 2-way ANOVA, *P* < 0.05). When comparing treated *vs.* untreated normoxic pregnancy, maternal treatment with allopurinol significantly decreased short telomere length in vascular tissue of adult offspring irrespective of age (Fig. 1*B* *vs*. Fig. 2*B*; 2-way ANOVA, *P* < 0.05). In contrast to untreated normoxic pregnancy, aged relative to young adult offspring of normoxic pregnancy treated with maternal allopurinol no longer showed a significant impairment in the femoral relaxant response to methacholine (Fig. 2*A*). Similarly, in contrast to untreated normoxic pregnancy, aged relative to young adult offspring of normoxic pregnancy treated with maternal allopurinol no longer showed a significant increase in the frequency of short telomeres in vascular tissue (Fig. 2*B*). However, they still showed a modest decrease in the frequency of long telomeres in vascular tissue (Fig. 2*E*). Compared with untreated hypoxic pregnancy, maternal treatment with allopurinol during hypoxic pregnancy restored femoral endothelial function in aged but not in young adult offspring (Fig. 2*A*), and this effect was associated with a lack of an increase in the frequency of short telomeres (Fig. 2B) and a significant, albeit modest increase in the frequency of long telomeres (Fig. 2*E*) in vascular tissue at 15 mo of age.

### Effects on inflammatory markers

**Figure 3.**
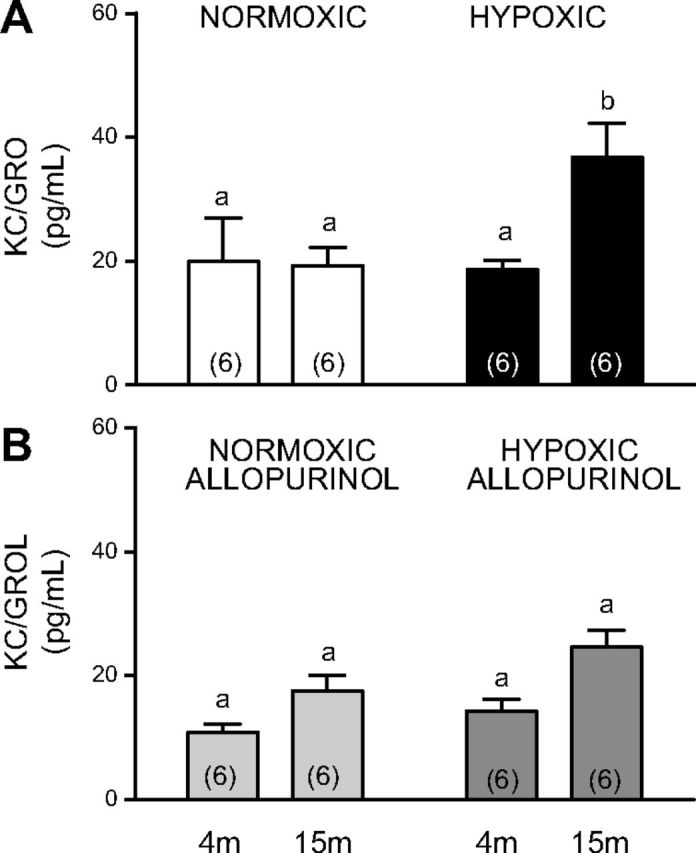
Effects of aging, developmental hypoxia and maternal allopurinol inflammatory markers. Values are means ± sem for the plasma concentration (pg/ml) of the proinflammatory chemokine KC/GRO in 4- and 15-mo-old offspring of untreated normoxic (white bars) or hypoxic (black bars) pregnancy (*A*) and in 4- and 15-mo-old offspring of normoxic (stippled bars) or hypoxic (dark gray bars) pregnancy treated with allopurinol (*B*). Bars with different letters are significantly (*P* < 0.05) different (2-way ANOVA and *post hoc* Tukey’s test).

Plasma concentrations of the inflammatory markers IFN-γ, IL-5, and C-reactive protein were similar in offspring of normoxic or hypoxic pregnancy at 4 and 15 mo of age (**Table 1**). However, the plasma concentration of the proinflammatory chemokine KC/GRO, the rodent equivalent of human IL-8 (41), was significantly elevated in aged offspring of hypoxic pregnancy relative to aged offspring of normoxic pregnancy (**Fig. 3**). Furthermore, maternal treatment with allopurinol prevented the increase in plasma KC/GRO in aged offspring of hypoxic pregnancy (Fig. 3).

**TABLE 1.** Pregnancy characteristics, offspring body weight and offspring inflammatory markers

Variable	N	H	HA	NA
Days of gestation	22.0 ± 0.1	22.1 ± 0.1	22.2 ± 0.1	22.0 ± 0.1
Litter size	13.4 ± 0.5	13.5 ± 0.5	12.5 ± 0.6	14.2 ± 0.5
Male:female ratio	0.9 ± 0.2	0.9 ± 0.2	1.0 ± 0.2	1.2 ± 0.2
Birth weight (g)	6.4 ± 0.3	6.4 ± 0.2	6.7 ± 0.1	6.3 ± 0.1
Weight (g)				
4 mo	568.3 ± 13.6	552.3 ± 12.5	556.2 ± 11.5	548.2 ± 11.1
15 mo	810.2 ± 21.3	767.1 ± 29.1	785.9 ± 26.4	801.4 ± 27.2
Inflammatory markers				
IFN γ (pg/ml)				
4 mo	11.09 ± 1.2	9.6 ± 0.8	9.2 ± 1.5	11.6 ± 0.7
15 mo	11.2 ± 1.2	12.0 ± 1.4	12.0 ± 1.7	12.0 ± 1.4
IL-5 (pg/ml)				
4 mo	88.0 ± 4.5	82.5 ± 3.3	78.3 ± 5.3	96.4 ± 4.7
15 mo	62.8 ± 6.6	63.2 ± 7.9	62.5 ± 5.4	73.9 ± 6.3
CRP (mg/L)				
4 mo	255.3 ± 16.4	243.6 ± 11.1	266.9 ± 12.3	235.9 ± 11.5
15 mo	279.4 ± 20.7	257.3 ± 14.2	279.2 ± 16.6	269.9 ± 11.2

Values are means ± sem for the duration of pregnancy (days of gestation), litter size, male: female pup sex ratio, birth weight and body weight at 4 and 15 mo, as well as plasma concentration of the inflammatory markers at 4 and 15 mo. Groups are normoxic or hypoxic pregnancy with (NA, *n* = 22; HA, *n* = 25) or without (*N*, *n* = 20; hypoxic H, *n* = 23) maternal treatment with allopurinol. Inflammatory markers were obtained in a subset of offspring of *n* = 6 in each group. CRP, C reactive protein ; H, hypoxic; HA, hypoxic allopurinol; N, normoxic; NA, normoxic allopurinol.

### Effects on pregnancy characteristics

There were no significant effects of hypoxic pregnancy or of allopurinol treatment on gestation length, litter size, fetal pup sex ratio, birth weight, or body weight at 4 and 15 mo of age (Table 1).

## DISCUSSION

The current findings show that aging in offspring of normoxic pregnancy promoted endothelial dysfunction in peripheral resistance circulations and that this effect was associated with shortening of telomere length in the vasculature. Maternal treatment with allopurinol in normoxic pregnancy protected the adult offspring against loss of endothelial function with aging. Developmental hypoxia programmed an earlier loss of endothelial function in peripheral resistance circulations in young adult offspring. However, maternal treatment with allopurinol in hypoxic pregnancy only rescued endothelial function in aged but not in young adult offspring. This protective effect of allopurinol on programmed endothelial dysfunction in aged but not in young adult offspring of hypoxic pregnancy was matched with the maintenance of vascular telomere length at 15 but not at 4 mo of age. In addition, 15-mo-old offspring of hypoxic pregnancy had significantly increased plasma levels of the proinflammatory chemokine KC/GRO. In contrast, this increase in plasma KC/GRO did not occur in 15-mo-old offspring of hypoxic pregnancy following maternal treatment with allopurinol. Collectively, the data partially support the hypotheses tested and reveal that developmental hypoxia leading to an earlier loss of endothelial function is not associated with accelerated vascular telomere shortening in young adult offspring. Furthermore, activation of the xanthine oxidase pathway and increased inflammation are mechanisms involved in promoting endothelial dysfunction in aged offspring of hypoxic pregnancy; therefore maternal treatment with allopurinol under these circumstances is protective against endothelial dysfunction. Conversely, accelerated loss of endothelial function in young adult offspring of hypoxic pregnancy occurs *via* mechanisms other than xanthine oxidase activation; under these conditions, maternal treatment with allopurinol is not protective.

Several studies have shown that aging promotes vascular stiffness (42) and vascular dysfunction (9) leading to decreased blood flow (43). Studies in humans and rodents show that vascular dysfunction and reduced blood flow with aging is associated with endothelial-dependent mechanisms (43–46). Aging decreases the ability of the endothelium to produce NO (45), due in part to enhanced production of reactive oxygen species and reduced expression of endothelial NO synthase, with consequent reduction in vascular NO bioavailability (44). Previous studies have also reported that vascular telomere length is inversely related with advancing age, with decreased telomere length thought to be associated with endothelial senescence in vascular dysfunction (15, 47, 48). Data in the present study showing that aging in offspring of normoxic pregnancy promoted endothelial dysfunction in peripheral resistance circulations and that this effect was associated with shortening of vascular telomere length are therefore consistent with the literature.

Independent studies have reported that chronic fetal hypoxia can program endothelial dysfunction in later life (7, 28, 49). Chronic fetal hypoxia, leading to a significant increase in fetal hematocrit, promotes fetal aortic wall thickening and increased oxidative stress in the fetal heart and vasculature by the end of gestation (28). In the fetal vasculature, increased generation of reactive oxygen species during hypoxic pregnancy react with NO decreasing its bioavailability (26, 28, 50–52). Chronic fetal hypoxia therefore promotes a sustained increase in the vascular oxidant tone leading to the abnormal development of endothelial function, in particular in peripheral resistance circulations (7, 12, 28). By adulthood, oxidative stress levels in the cardiovascular system between offspring of normoxic or hypoxic pregnancy are not different. However, chronic fetal hypoxia sets functional deficits in both the heart and the peripheral circulation of the adult offspring (28). In the peripheral circulation, this is reflected by NO-dependent endothelial dysfunction (28). These adverse effects of hypoxic pregnancy on the cardiovascular system are prevented by maternal treatment with antioxidants such as vitamin C (27, 28, 53) or resveratrol (30, 54), suggesting a role for oxidative stress in the developmental programming of endothelial dysfunction. As aging also involves oxidative stress (22–25, 55), several studies have suggested that developmental programming of cardiovascular dysfunction by adverse intrauterine conditions may be a form of accelerated aging (16–21). In support of this idea, past (13) and present data show that developmental hypoxia programmed an earlier loss of endothelial function in peripheral resistance circulations in young adult offspring. Furthermore, rodent models of maternal under- (16) as well as overnutrition (56) program accelerated cardiovascular degeneration with normal aging with reductions in telomere length and in life span (18). However, additional data in the present study show that the effect of developmental hypoxia on endothelial function was not associated with accelerated vascular telomere shortening in young adult offspring. These data therefore oppose the idea that programming of vascular dysfunction by developmental hypoxia is just a form of accelerated vascular aging, adding new conceptual insight to the fields of aging and of developmental programming. The data also highlight that different suboptimal conditions during pregnancy may program cardiovascular dysfunction in the adult offspring *via* several mechanisms.

The purine analog allopurinol is a known inhibitor of xanthine oxidase (37), a powerful pro-oxidant mechanism stimulated by chronic hypoxia primarily due to accumulation of the substrate hypoxanthine (35, 57). Indeed, there is evidence of increased xanthine oxidase expression in vascular endothelial cells in response to hypoxia (58). Additionally, increased xanthine oxidase activity is associated with excessive generation of reactive oxygen species, along with the induction of oxidative stress and of cardiovascular dysfunction, and treatment with allopurinol is protective in cardiovascular disease states (59–63). We have previously reported that daily ingestion of allopurinol by the pregnant rat at the dosing regimen used in this study not only crossed the placenta, but also promoted inhibition of xanthine oxidase within maternal, placental, and fetal tissues (37). In the present study, we show that maternal treatment with allopurinol has the pronounced effect to shift the population distribution of different lengths of telomeres toward a reduction in short telomere length in vascular tissue of adult offspring of normoxic or hypoxic pregnancy. Furthermore, we show that maternal treatment with allopurinol in hypoxic pregnancy rescued endothelial function but only in aged and not in young adult offspring. This protective effect of allopurinol on programmed endothelial dysfunction in aged but not in young adult offspring of hypoxic pregnancy was matched with the maintenance of vascular telomere length at 15 but not at 4 mo of age. Combined, therefore, the data show that although prenatal treatment with the xanthine oxidase inhibitor allopurinol does not protect against endothelial dysfunction in young adult offspring of hypoxic pregnancy, it does in aged offspring of normoxic or of hypoxic pregnancy. Therefore, activation of the xanthine oxidase pathway is a mechanism involved in promoting endothelial dysfunction in aged offspring independent of normoxic or hypoxic pregnancy. Conversely, accelerated loss of endothelial function in young adult offspring of hypoxic pregnancy occurs *via* mechanisms other than activation of xanthine oxidase. The lack of association between accelerated endothelial dysfunction and accelerated vascular telomere shortening in young adult offspring of hypoxic pregnancy, coupled with a protective effect of allopurinol only in aged offspring of either normoxic or hypoxic pregnancy but not in young adult offspring of hypoxic pregnancy, are findings that again suggest that programming of endothelial dysfunction in adulthood by developmental hypoxia is not just a form of accelerated aging. Furthermore, vascular aging in the form of endothelial dysfunction involves oxidative stress derived in part from activation of xanthine oxidase even prior to birth and maternal treatment with allopurinol during pregnancy may protect against vascular aging in the adult offspring irrespective of normoxic or hypoxic pregnancy.

Aging has been associated with shortening of telomere length and increased inflammatory markers (9, 64, 65). Therefore, in the present study, plasma samples obtained from young adult and aged offspring of normoxic or hypoxic pregnancy were processed for circulating indices of inflammation. Aged offspring of hypoxic pregnancy had significantly increased plasma levels of the proinflammatory chemokine KC/GRO. In contrast, this increase in plasma KC/GRO was significantly diminished in aged offspring of hypoxic pregnancy with maternal allopurinol treatment. Increased vascular expression of KC/GRO has been reported to be central to the progression of vascular aging (66). Therefore, protection against inflammation provides an additional mechanism for the maintenance of vascular telomere length during aging in hypoxic pregnancy treated with allopurinol.

Finally, data in the present study also show that hypoxic pregnancy or allopurinol treatment did not affect the pregnancy characteristics, birth weight, or body weight at 4 or 15 mo of age. Although several models of more severe (10%) hypoxic pregnancy in rodents in the last third of gestation do induce a decrease in birth weight (10, 13, 32, 49), this rodent model of developmental hypoxia is different as it is milder (13%) and is early in onset, starting at d 6 of gestation. Because of the temporal pattern of placental growth during gestation (67), early onset hypoxic pregnancy permits placental compensation (68). This protects fetal growth despite hypoxic pregnancy, yielding a normal birth weight (28, 53). This finding is also important because the literature has previously linked fetal growth restriction and postnatal accelerated growth with the greatest increased risk of cardiovascular disease in later life (69). Therefore, the present data underline that neither intrauterine growth restriction nor alterations in postnatal growth are necessary prerequisites for endothelial dysfunction in later life programmed by adverse intrauterine conditions.

In conclusion, the data show that vascular aging involves xanthine oxidation activation even prior to birth and that maternal treatment with the xanthine oxidase inhibitor allopurinol during pregnancy can decelerate vascular aging in the offspring. The data also provide the first evidence, to our knowledge, that premature endothelial dysfunction programmed by developmental hypoxia is different from accelerated vascular aging, that it does not involve xanthine oxidase activation and thereby it requires different treatment.

## Abbreviations

KC/GRO: keratinocyte chemoattractant/growth-regulated oncogene
